# Type III Effectors of *Pseudomonas syringae* pv. *tomato* DC3000: Molecular Mechanisms, Host Manipulation Networks, and Implications for Plant Disease Resistance

**DOI:** 10.3390/life16101757

**Published:** 2026-10-20

**Authors:** Xiaoju Zhong, Jianzhong Huang

**Affiliations:** Department of Basic Medicine, Fuzhou Medical College, Fuzhou 344000, China; xiaojuzhong1983@163.com

**Keywords:** *Pseudomonas syringae* pv. *tomato* DC3000, type III secretion system, type III effectors, effectorome, plant immunity, host manipulation, disease resistance, CRISPR, bacterial virulence

## Abstract

*Pseudomonas syringae* pv. *tomato* DC3000 (*Pst* DC3000) is a reference system for dissecting how type III-secreted effectors (T3Es) suppress plant immunity and reconfigure host physiology. Here, we reassess the DC3000 effector repertoire through an evidence-graded framework that links experimentally resolved effector–target mechanisms to repertoire-level genetic interactions and, ultimately, to disease-resistance strategies. We distinguish findings demonstrated with *Pst* DC3000 from mechanistic examples derived from other *P. syringae* pathovars and from computationally generated hypotheses. At the molecular level, validated DC3000 effectors perturb pattern-recognition receptor signaling, protein turnover, vesicle trafficking, plasmodesmatal communication, organelle function, and hormone-associated processes. At the repertoire level, deletion and reconstruction studies reveal functional redundancy, specialization, conditional suppression, and context-dependent interactions; importantly, convergence on a common host phenotype is not treated as evidence of direct physical cooperation between effectors. We further evaluate how effectoromics, multi-omics, single-cell and spatial approaches, structure prediction, and machine-learning tools can prioritize new hypotheses while emphasizing their need for genetic, biochemical, and infection-based validation. Finally, we consider how conserved effector targets, susceptibility factors, NLR engineering, and CRISPR-based editing may translate mechanistic knowledge into more durable bacterial-disease resistance. This synthesis positions the DC3000 system as a bridge between mechanistic effector biology, quantitative effector-network analysis, and experimentally testable strategies for crop protection.

## 1. Introduction

Plants are constantly challenged by diverse microbial communities, encompassing both beneficial microorganisms and potential pathogens. To maintain cellular homeostasis and survive microbial attacks, plants have evolved multilayered immune systems that perceive invading microbes and activate coordinated defense responses [1,2]. Pattern-triggered immunity (PTI) is initiated by the recognition of conserved microbial-associated molecular patterns (MAMPs) through cell surface pattern recognition receptors (PRRs), whereas effector-triggered immunity (ETI) is activated when intracellular nucleotide-binding leucine-rich repeat (NLR) proteins detect pathogen-derived effectors [3,4]. Rather than operating as independent defense systems, PTI and ETI represent interconnected immune layers that collectively shape the outcome of plant–microbe interactions [4,5].

Plant pathogenic bacteria have evolved sophisticated mechanisms to overcome these immune barriers, among which the type III secretion system (T3SS)-mediated delivery of type III effectors (T3Es) represents a central virulence strategy [5,6]. Through a syringe-like secretion apparatus, bacterial pathogens inject T3Es directly into host cells, where these proteins manipulate immune signaling, cellular organization, and metabolic processes to create a cellular environment favorable for infection [5,6,7]. Unlike conventional virulence factors that primarily cause host damage, T3Es function as molecular regulators capable of rewiring host cellular states and redirecting endogenous processes to support pathogen survival. Understanding how bacterial effectors reshape host biology has therefore become a fundamental question in plant–microbe interaction research [7,8].

Among phytopathogenic bacteria, *Pst* DC3000 has become one of the best-established model systems for investigating T3E-mediated host manipulation [9]. As the causal agent of bacterial speck disease in tomato (*Solanum lycopersicum*), *Pst* DC3000 also infects the model plant *Arabidopsis thaliana*, providing a genetically tractable pathosystem for dissecting bacterial virulence and plant immunity [9,10]. The availability of its complete genome sequence, together with extensive genetic resources and well-developed experimental systems, has greatly facilitated functional characterization of its T3E repertoire [10,11]. Approximately 30 functional T3Es, predominantly belonging to the Hop and Avr families, have been identified in *Pst* DC3000, enabling systematic investigation of how bacterial effectors manipulate host cellular processes and suppress immune responses [11,12,13].

Early work on *Pst* DC3000 established a series of experimentally tractable effector–target relationships that still anchor the field. AvrPto interferes with pattern-recognition receptor (PRR) kinases, AvrPtoB combines receptor-associated interference with E3 ubiquitin-ligase activity, HopM1 destabilizes the ARF-GEF MIN7 and perturbs vesicle trafficking, HopI1 remodels chloroplast functions, and HopF2 inhibits MAPK signaling [14,15,16,17,18]. These studies defined distinct biochemical entry points into host immunity and cell biology. They also exposed an important limitation of the one-effector–one-target view: the virulence contribution measured for any one effector depends on the remainder of the bacterial repertoire and on the host in which that activity is assayed. That dependence became explicit in effector-deletion and reconstruction experiments. DC3000 polymutants revealed extensive functional overlap, whereas highly disarmed strains showed that selected effector combinations can restore substantial pathogenic activity [12,19]. Pairwise delivery experiments further demonstrated that one effector can suppress or modify the host response elicited by another [20,21]. These findings justify a network perspective, but they also require careful terminology: genetic compensation, convergence on the same host process, conditional suppression, and direct molecular interaction are different forms of evidence and should not be conflated.

**Figure 1:**
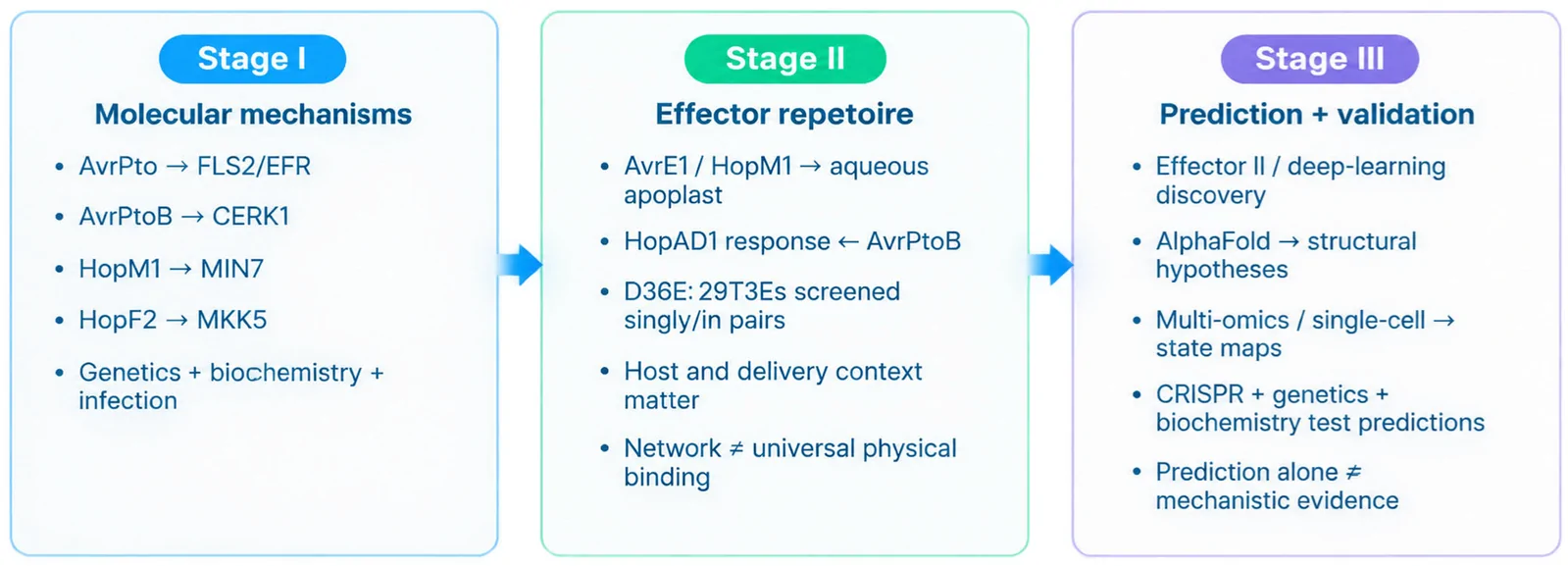
Evidence progression in *Pst* DC3000 effector research. Stage I summarizes experimentally resolved effector–target mechanisms. Stage II adds repertoire-level genetic and functional relationships, including redundancy, conditional suppression, and context dependence. Stage III integrates computational discovery, structure prediction, multi-omics, and single-cell analyses with explicit experimental validation. Arrows indicate an increase in integration, not a replacement of causal experiments; computational predictions are treated as hypotheses until validated. Representative mechanistic and repertoire-level evidence is supported by refs. [12,14,15,16,17,18,19,20,21,34], and representative computational approaches by refs. [26,27,28,29,30,31,32,33].

New technologies have broadened the questions that can be asked of this system. Multi-omics and single-cell approaches resolve infection-associated host states; structural prediction can reveal remote relationships and candidate interaction surfaces; and machine-learning models can prioritize candidate T3Es from genomic data [22,23,24,25,26,27,28,29,30,31,32,33]. None of these approaches, however, converts correlation or model confidence into mechanism. Their value is greatest when predictions are tied back to perturbation experiments, native T3SS delivery, biochemical validation, and quantitative infection phenotypes. Accordingly, this review focuses on three connected questions: which host processes are manipulated by experimentally characterized *Pst* DC3000 T3Es; what forms of genetic or functional interaction are actually supported within the DC3000 repertoire; and how can these mechanistic and network-level insights inform plant disease resistance? We use DC3000-specific evidence whenever available, identify observations derived from other *P. syringae* pathovars, and separate experimentally established relationships from computationally inferred ones. The resulting framework links molecular mechanism, repertoire organization, predictive technologies, and translational plant pathology (Figure 1).

Notably, several influential reviews have addressed *Pst* DC3000 pathogenicity, T3SS regulation, effector repertoire evolution, or broader effector-network concepts [9,33,34,35,36,37]. The present review is narrower in organismal focus but more explicit in evidence integration. Rather than treating “network” as a general metaphor, we connect defined DC3000 effector–target mechanisms to deletion/reconstruction genetics, pairwise effector effects, host-cell-state remodeling, and disease-resistance implications. Table 1 summarizes how this emphasis differs from selected prior reviews and network-focused studies. This is a focused narrative review rather than a systematic review or meta-analysis. Literature was surveyed through PubMed, Web of Science Core Collection, Scopus, and Google Scholar, with emphasis on studies available through September 2026. Searches combined terms including “*Pseudomonas syringae*”, “DC3000”, “type III effector”, “T3SS”, “effectorome”, “host target”, “virulence”, “single-cell”, “multi-omics”, “AlphaFold”, “machine learning”, “CRISPR”, and “disease resistance”. Primary studies using *Pst* DC3000 and defined plant hosts were prioritized for mechanistic claims; work from other pathovars was included when it provided a clearly labeled mechanistic comparator, and recent reviews were used principally to frame field-level developments.

## 2. The T3SS Machinery and the Dynamic Organization of the *Pst* DC3000 Effector Repertoire

### 2.1. The T3SS as a Regulatory Platform for Coordinated Effector Deployment

The T3SS represents a highly conserved and specialized protein delivery machinery employed by Gram-negative bacterial pathogens to directly manipulate host cellular processes. In plant pathogenic *P. syringae*, the T3SS consists of a membrane-associated secretion apparatus, an extracellular Hrp pilus, and a translocation complex that together enable the delivery of bacterial effectors across the bacterial envelope, plant cell wall, and host plasma membrane [39,40,41,42]. In *Pst* DC3000, the core components of the T3SS are encoded within the *hrp*/*hrc* pathogenicity island, which also contains conserved and variable regions associated with effector secretion and diversification [40]. The functionality of this secretion system is essential for efficient effector translocation, bacterial multiplication, and disease development in susceptible hosts [39,40,41,42].

Beyond functioning as a physical delivery apparatus, the T3SS serves as a tightly regulated platform that coordinates the production and deployment of bacterial effectors during infection. Expression of T3SS structural genes and numerous effector genes is controlled by the HrpR/HrpS–HrpL regulatory cascade, in which the alternative sigma factor HrpL activates transcription of genes containing conserved *hrp*-box promoter elements [43]. This regulatory system integrates multiple environmental and host-derived signals, including nutrient availability, bacterial population density, carbon sources, and plant-associated metabolites, allowing *Pst* DC3000 to optimize effector expression according to infection conditions [43,44,45,46]. In particular, host-derived acidic amino acids, such as aspartate and glutamate, can activate the AauS–AauR signaling system and contribute to induction of T3SS-associated genes [46].

Therefore, T3SS-mediated pathogenicity is not solely determined by the biochemical activities of individual effectors, but also depends on the coordinated regulation of effector expression, secretion timing, and deployment strategies. Such regulatory coordination provides the foundation for the functional integration of multiple effectors during host colonization.

### 2.2. Diversity and Functional Architecture of the Pst DC3000 Effector Repertoire

Genome-wide analyses have revealed that *Pst* DC3000 contains one of the most extensively characterized repertoires of type III effectors among plant pathogenic bacteria. Early studies identified approximately 53 *hop*/*avr* genes and pseudogenes, among which approximately 30 were predicted to encode functional or actively expressed T3Es. Subsequent functional investigations have generally supported the presence of a core repertoire consisting of approximately 28–30 effectors [11,34,47]. These effectors mainly belong to the Hop and Avr families and exhibit substantial diversity in sequence characteristics, biochemical activities, intracellular localization, and host targets [11,34,47].

The diversity of the *Pst* DC3000 effector repertoire provides bacteria with the capacity to manipulate multiple aspects of host biology. Individual effectors have been shown to interfere with immune receptor signaling, intracellular trafficking, protein degradation, organelle functions, and hormone-associated regulatory pathways [34,47]. However, the effector repertoire should not be viewed as a simple collection of independent virulence factors. Instead, these effectors exhibit a functional organization in which distinct proteins frequently converge on shared host processes while maintaining specialized molecular activities.

Comparative and functional analyses suggest that some conserved effectors target fundamental regulatory hubs required for successful infection, whereas more variable effectors contribute to host adaptation and immune evasion [34,47]. This combination of conserved functional modules and diversified activities enables *Pst* DC3000 to maintain pathogenic fitness under different host conditions. Thus, the composition of the effector repertoire reflects not only the accumulation of virulence-associated proteins but also an evolutionary strategy for constructing a flexible host manipulation system.

### 2.3. Evolutionary Diversification Shapes Effector Network Plasticity

The diversity of *Pst* DC3000 effectors reflects an ongoing evolutionary conflict between bacterial adaptation and host immune surveillance. Although T3Es promote bacterial infection by suppressing host defenses and modifying cellular processes, individual effectors may also become recognized by plant immune receptors, particularly NLR proteins, resulting in ETI [48,49]. Therefore, effector evolution is constrained by the need to maintain virulence-associated functions while avoiding host recognition.

Multiple evolutionary mechanisms contribute to diversification of the *Pst* DC3000 effector repertoire, including sequence variation, positive selection, gene gain and loss, horizontal gene transfer, recombination, and pseudogenization [35,48,49,50]. Comparative genomic analyses have demonstrated that closely related *P. syringae* lineages may possess substantially different effector complements, contributing to differences in host range and pathogenic potential [35,48,50]. Importantly, evolutionary diversification does not simply increase the number of effectors but reshapes the functional organization of effector systems. Sequence diversification may alter host recognition, generate new biochemical activities, or modify interactions among existing effectors, thereby influencing the overall architecture of bacterial virulence strategies. Consequently, *Pst* DC3000 T3Es should be considered dynamic components of an evolving effector network rather than a fixed group of independent virulence determinants.

### 2.4. Functional Organization of the Effector Repertoire: Redundancy and Specialization

A defining characteristic of the *Pst* DC3000 effector repertoire is the coexistence of functional redundancy and specialization. Redundancy allows bacteria to preserve pathogenic capacity when individual effectors are lost, mutated, or recognized by host immunity. Multiple effectors may therefore converge on similar host processes and compensate for each other’s loss [34,47,51]. Experimental evidence supporting this concept has been obtained through analysis of effector deletion mutants and reconstructed bacterial strains. For example, HopAA1-1 exhibits functional overlap with other virulence-associated factors during bacterial speck development [51]. Similarly, studies using disarmed *Pst* DC3000 strains demonstrated that a subset of effectors can restore major aspects of bacterial pathogenicity, indicating extensive functional buffering within the effector repertoire [34].

However, redundancy does not indicate complete functional equivalence. Different effectors possess distinct biochemical properties and contribute to infection through specialized mechanisms. AvrPto and AvrPtoB primarily interfere with early immune perception and signaling, whereas HopM1 and AvrE1 contribute to remodeling of the host apoplastic environment [34]. Furthermore, effector activities can depend on the presence of other effectors, as demonstrated by interactions in which AvrPtoB suppresses immune-associated cell death induced by HopAD1 [21]. Thus, redundancy provides robustness, whereas specialization expands the regulatory capacity of the effector repertoire. Together, these organizational principles allow *Pst* DC3000 to simultaneously manipulate multiple host processes and maintain infection efficiency under diverse environmental and immune conditions.

### 2.5. From Effector Repertoire to Integrated Virulence Networks

Genetic and physiological evidence supports treating the *Pst* DC3000 effector repertoire as a context-dependent virulence system rather than a list of independent proteins [12,19,20,21]. In this review, “network” is used operationally for activities linked by genetic compensation, conditional suppression, or convergence on connected host processes; it does not imply that all effectors interact physically. Specific evidence for redundancy, specialization, pairwise antagonism, and repertoire-level interplay is evaluated in Section 4.

## 3. Type III Effectors as Modulators of Plant Immune Responses and Cellular States

**Figure 2:**
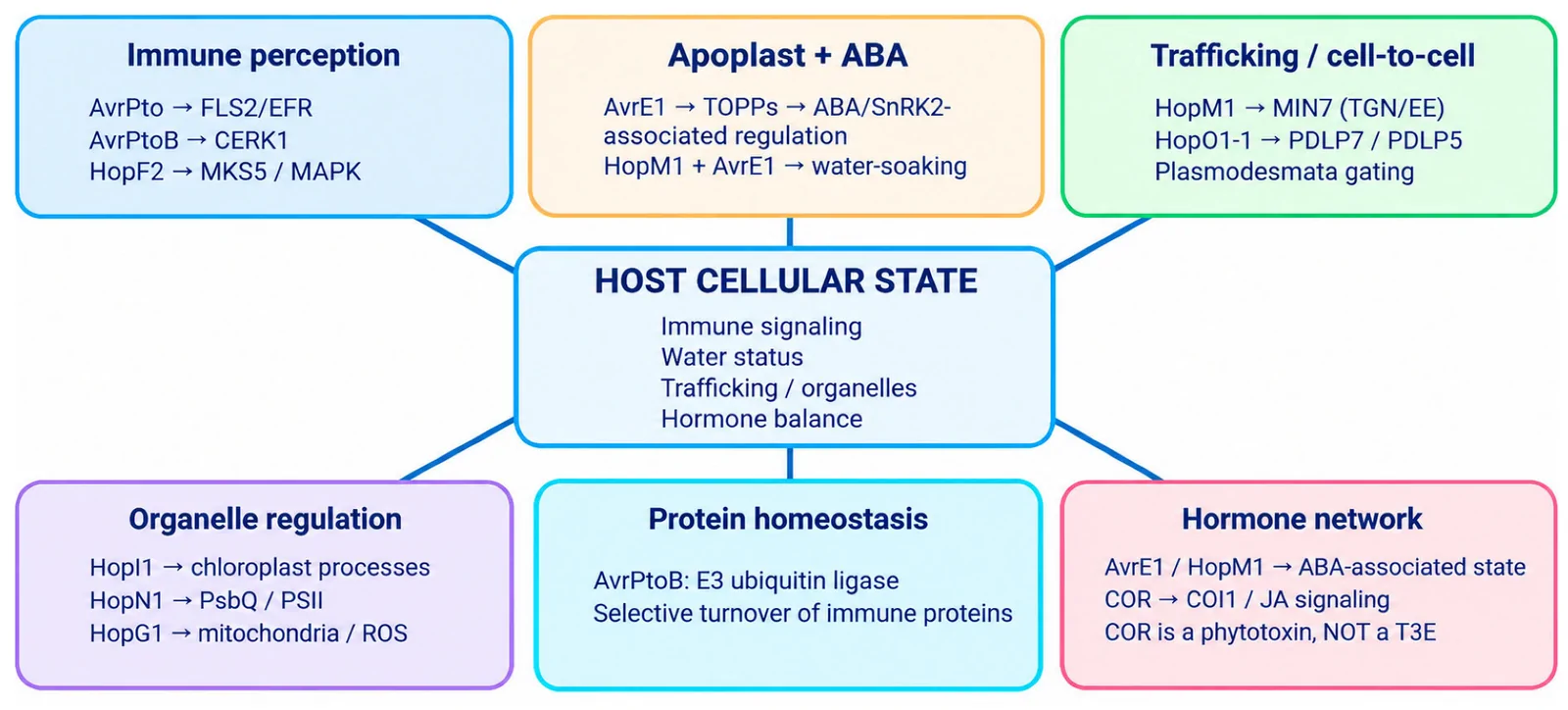
Experimentally anchored host processes manipulated during *Pst* DC3000 infection. The diagram links representative DC3000 T3Es to validated targets or host processes: AvrPto–FLS2/EFR, AvrPtoB–CERK1/protein turnover, HopF2–MKK5/MAPK signaling, AvrE1–TOPP/ABA-associated water regulation, HopM1–MIN7 trafficking, HopO1-1–plasmodesmatal proteins, and organelle-associated activities of HopI1, HopN1, and HopG1. Coronatine (COR) is displayed separately because it is a phytotoxin rather than a T3SS-secreted effector. Convergence on a common physiological outcome does not by itself demonstrate direct effector–effector binding. Representative experimentally supported relationships are summarized from refs. [14,15,16,17,18,52,53,54,55,56,57,58,59,60,61,62,63].

T3Es are central determinants of the pathogenic lifestyle of *Pst* DC3000. Rather than acting as simple virulence factors that directly damage host tissues, T3Es function as sophisticated molecular regulators that redirect host cellular processes toward conditions favorable for bacterial survival and proliferation. Following translocation into plant cells, these effectors interfere with multiple layers of host regulation, including immune perception, signal transduction, intracellular trafficking, organelle function, hormone homeostasis, and protein stability. Although individual T3Es often possess distinct biochemical activities, their biological effects are rarely restricted to a single pathway. Increasing evidence indicates that *Pst* DC3000 pathogenicity is determined by coordinated interactions among multiple effectors that target interconnected host processes (Figure 2). Thus, understanding T3E function requires moving beyond individual effector–target relationships toward a broader view of how bacterial proteins reshape host cellular states during infection.

### 3.1. Suppression of Plant Immune Perception and Early PTI Signaling

The first barrier encountered by bacterial pathogens is the plant immune surveillance system, which detects conserved microbial signatures through cell-surface PRRs. To establish infection, *Pst* DC3000 deploys multiple effectors that interfere with receptor activation, downstream kinase signaling, and immune amplification pathways.

AvrPto represents one of the best-characterized examples of an immune-targeting effector. AvrPto interacts with the kinase domains of PRRs such as FLS2 and EFR and suppresses receptor-mediated immune signaling [14]. Subsequent analyses further indicated that, although BAK1 is an essential co-receptor for several PRRs, it is not a direct in planta target of AvrPto; instead, AvrPto-mediated inhibition of FLS2 prevents phosphorylation of downstream components such as BIK1 [52]. In resistant tomato genotypes, however, AvrPto is recognized through the Pto/Prf immune receptor complex, illustrating the evolutionary constraint imposed on bacterial effectors, which must retain virulence activity while avoiding host surveillance. AvrPtoB extends this strategy by combining immune signaling interference with manipulation of host protein stability. Its C-terminal domain possesses E3 ubiquitin ligase activity that is required for efficient suppression of plant immunity [53]. AvrPtoB can target immune-associated receptor kinases for ubiquitination and degradation, including CERK1, thereby directly linking PRR suppression to host proteostasis [54]. Thus, AvrPtoB functions as a multidomain effector capable of suppressing immunity through both receptor-associated interactions and selective protein turnover.

Other effectors interfere with signaling downstream of PRRs. HopF2 functions as an ADP-ribosyltransferase that interacts with and modifies *Arabidopsis* MAP kinase kinases, particularly MKK5, thereby inhibiting MAPK activation and downstream PTI responses [18]. HopAO1 acts at an earlier level by functioning as a protein tyrosine phosphatase that reduces phosphorylation of the EF-Tu receptor EFR and consequently suppresses EFR-dependent immune signaling [55]. HopA1 provides an informative example of how effector sequence diversification can alter immune recognition. The HopA1 variant from *P. syringae* pv. syringae 61 is recognized by the *Arabidopsis* TIR-NBS-LRR receptor RPS6, whereas the divergent HopA1 protein encoded by *Pst* DC3000 does not activate RPS6-mediated resistance. The two proteins share only approximately 57% amino acid identity, illustrating how effector diversification can permit retention of related proteins while altering recognition by host immune receptors [56].

Collectively, these examples demonstrate that *Pst* DC3000 does not simply suppress isolated immune components. Instead, its effectors target multiple regulatory nodes spanning PRR activation, receptor phosphorylation, MAPK signaling, and protein stability.

### 3.2. Remodeling of Apoplastic Environments and Intracellular Trafficking

Successful bacterial colonization requires not only suppression of immune signaling but also remodeling of the host environment in which bacterial populations proliferate. *Pst* DC3000 effectors contribute to this process by modifying apoplastic water availability, vesicle trafficking, and intercellular communication.

AvrE1 and HopM1 represent particularly important determinants of apoplastic remodeling. Genetic and physiological studies show that these effectors functionally converge on establishment of an aqueous apoplast favorable for bacterial proliferation [57]. Both effectors promote ABA-associated transcriptional reprogramming, stomatal closure, reduced transpiration, and water-soaking, thereby converting the normally air-filled leaf apoplast into a more permissive aqueous environment [57]. Independent mechanistic analyses further showed that AvrE directly targets *Arabidopsis* type one protein phosphatases (TOPPs), activating ABA signaling through the SnRK2 pathway and promoting stomatal closure and water-soaking [58]. HopM1 additionally perturbs the endomembrane system by promoting degradation of the *Arabidopsis* ARF-GEF MIN7, an immunity-associated regulator localized to the trans-Golgi network/early endosome [16,59]. MIN7 contributes to vesicle formation, cargo recycling, and membrane-trafficking processes required for PTI- and ETI-associated defense. Its loss therefore provides a mechanistic link between effector activity and the host’s capacity to mobilize membrane-associated defense functions. Importantly, this trafficking phenotype is experimentally distinct from the water-status effects shared by HopM1 and AvrE1, even though both activities contribute to successful colonization.

A second level of trafficking control occurs at plasmodesmata. HopO1-1 localizes to plasmodesmata, promotes intercellular molecular movement, and destabilizes the plasmodesmata-localized proteins PDLP7 and, potentially, PDLP5 [60]. This activity interferes with immune-associated plasmodesmatal closure and illustrates that DC3000 can alter not only intracellular vesicle traffic but also cell-to-cell connectivity. The distinction matters mechanistically: MIN7-dependent endomembrane trafficking and PDLP-dependent plasmodesmatal gating operate at different spatial scales and should not be collapsed into a single generic “trafficking” mechanism.

Taken together, the evidence supports a layered remodeling of the infection niche. AvrE1 and HopM1 alter apoplastic hydration; HopM1 perturbs MIN7-dependent endomembrane trafficking; and HopO1-1 modifies plasmodesmatal communication [57,58,59,60]. These processes are functionally connected at the level of tissue colonization, but current data do not require direct molecular cooperation among the effectors themselves.

### 3.3. Manipulation of Protein Homeostasis and Organelle Functions

Once delivered into host cells, *Pst* DC3000 effectors extensively remodel intracellular regulatory systems. Protein homeostasis represents one important target of bacterial manipulation, as illustrated by AvrPtoB-mediated ubiquitination and degradation of immune-associated proteins [53,54]. However, effector activity also extends to major host organelles that integrate metabolism, redox regulation, and immune signaling.

HopI1 provides a well-characterized example of chloroplast manipulation. HopI1 localizes to chloroplasts, remodels thylakoid architecture, suppresses salicylic acid accumulation, and contains a functional J domain that is required for its virulence activity [17]. These findings suggest that HopI1 exploits chloroplast Hsp70-associated processes to alter both chloroplast physiology and plant defense. HopN1 targets a distinct chloroplast-associated mechanism. This cysteine protease effector interacts with PsbQ, a component of the oxygen-evolving complex of photosystem II, and promotes its degradation. HopN1-mediated disruption of PsbQ reduces photosystem II activity and suppresses defense-associated ROS production and callose deposition [61]. Thus, manipulation of photosynthetic machinery can directly influence immune output.

Mitochondria represent another effector target. HopG1 from *Pst* DC3000 localizes to mitochondria, suppresses PTI and ETI-associated responses, reduces respiration, and alters cellular ROS homeostasis [62]. HopG1 has also been linked to reorganization of the actin cytoskeleton during DC3000 infection [63], illustrating how perturbation of organelle-associated processes can propagate to broader changes in cellular organization.

Together, these effectors demonstrate that *Pst* DC3000 preferentially targets cellular compartments that act as regulatory hubs. Manipulation of protein turnover, chloroplast function, photosynthetic electron transport, mitochondrial physiology, and redox homeostasis enables bacterial effectors to simultaneously influence immunity, metabolism, and cellular stress responses.

### 3.4. Regulation of Hormone Signaling and Defense–Growth Trade-Offs

Hormone signaling is another major layer of host regulation altered during *Pst* DC3000 infection. Because jasmonate (JA), salicylic acid (SA), and abscisic acid (ABA) influence immunity, stomatal behavior, water relations, and growth, bacterial manipulation of these pathways can reshape both defense output and the physical infection environment (Figure 2).

Coronatine (COR) is central to this discussion but must be distinguished from the T3E repertoire: COR is a diffusible bacterial phytotoxin, not a T3SS-secreted protein effector. It mimics the bioactive jasmonate conjugate JA-Ile, activates COI1-dependent JA signaling, promotes stomatal reopening, and engages the MYC2–ANAC019/ANAC055/ANAC072 cascade that lowers SA accumulation [64]. In parallel, the bona fide DC3000 T3Es AvrE1 and HopM1 promote ABA-associated transcriptional and physiological changes that favor water-soaking [57,58]. Thus, COR and T3Es converge on hormone-regulated host physiology through distinct delivery routes and molecular mechanisms.

Mechanistic examples from other *P. syringae* pathovars further show that protein effectors can directly manipulate the core JA machinery. HopZ1a from *P. syringae* pv. *syringae* acetylates JAZ repressors and promotes JA signaling [65], but canonical *Pst* DC3000 does not encode an endogenous HopZ1a [11,12,13]. HopX1 from *P. syringae* pv. *tabaci* 11528 is a cysteine protease that promotes JAZ degradation [66]. DC3000 carries HopX1 (PSPTO1988), a HopX1-family effector represented in standard repertoire inventories [11,12,13], yet the JAZ-cleavage mechanism in reference [66] was demonstrated with the *P. syringae* pv. *tabaci* 11528 allele; that activity should not be assigned to the DC3000 allele without allele-specific validation. These examples are therefore used as comparative mechanisms, not as evidence that the same biochemical reactions occur in DC3000. This separation of evidence avoids a common nomenclature problem. For DC3000, the strongest direct evidence in this section concerns COR-mediated JA signaling and AvrE1/HopM1-associated ABA and water relations; HopZ1a and the characterized *Pta*11528 HopX1 allele provide broader *P. syringae* comparators. The shared lesson is that bacterial virulence programs can reweight JA–SA–ABA signaling, but strain identity and experimental host must remain explicit when mechanistic conclusions are transferred across pathovars.

### 3.5. Integrated View: Effectors Remodel Host Cellular States Rather than Single Defense Pathways

The experimentally characterized DC3000 repertoire acts at multiple regulatory layers, but “cellular-state remodeling” is best regarded as a synthesis of measured mechanisms rather than a single experimentally observable entity. Individual T3Es alter receptor signaling, phosphorylation, protein stability, vesicle trafficking, plasmodesmatal communication, organelle physiology, and hormone-associated processes. Their combined consequences can change tissue hydration, immune output, redox state, and metabolic allocation (Table 2).

Highly disarmed DC3000 strains provide the clearest bridge from individual mechanisms to repertoire function. Reduced effector sets can recover substantial pathogenic activity, demonstrating functional buffering and division of labor [19,34]. AvrE1 and HopM1 both contribute to an aqueous apoplast [57,67], whereas pairwise screens in an effectorless background reveal suppression and enhancement relationships that are not predictable from single-effector phenotypes [20]. These data support network-level analysis, but they do not justify labeling every shared physiological effect as direct molecular cooperation. We therefore use an evidence-tiered network model: validated effector–host targets form the molecular layer; deletion/reconstruction phenotypes define redundancy or complementarity; pairwise experiments identify conditional effector–effector effects; and omics or computational analyses generate hypotheses about broader host-state organization. This hierarchy preserves mechanistic precision while allowing the repertoire to be analyzed as a system.

## 4. Functional Interactions and Effector Networks: Evidence from Redundancy, Specialization, and Context Dependence

**Figure 3:**
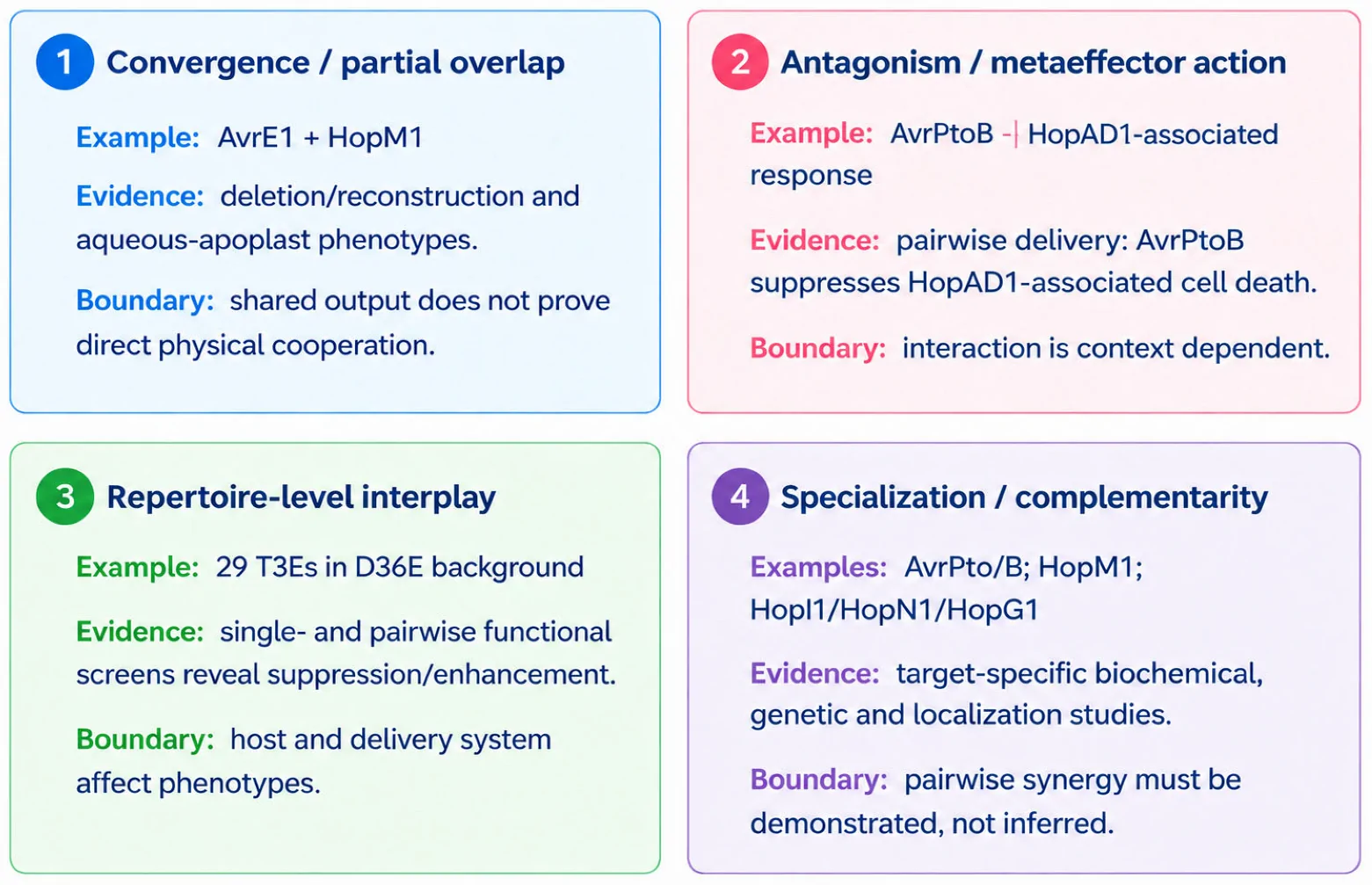
Evidence-defined organization of the *Pst* DC3000 effector repertoire. Four relationship classes are separated by their experimental basis: functional convergence/partial overlap, antagonistic or metaeffector action, repertoire-level interplay, and specialization/complementarity. AvrE1–HopM1 illustrates convergence on an aqueous apoplast; HopAD1–AvrPtoB provides a defined antagonistic interaction; and single/pairwise delivery of the 29-effector repertoire in a disarmed DC3000 background demonstrates broader context-dependent interplay. “Network” is used operationally and does not imply direct molecular interaction for every linked pair. Representative evidence for these relationship classes is provided in refs. [12,19,20,21,34,38,57,58,59,67].

The term “effector network” is useful only when the underlying relationship is specified. For *Pst* DC3000, the strongest evidence comes from four experimental classes: deletion/reconstruction phenotypes that reveal redundancy or buffering; target-specific studies that define specialization; pairwise delivery experiments that reveal conditional suppression or enhancement; and repertoire-scale screens that expose context dependence [12,19,20,21]. Section 4 therefore organizes network claims by evidence type rather than treating all co-occurring effector activities as equivalent (Figure 3).

### 4.1. Functional Redundancy Provides Robustness to Bacterial Infection

Functional redundancy is most directly inferred when removal of one component has little effect but combined deletions or reconstruction experiments uncover a shared virulence requirement. This pattern is prominent in DC3000: many single-effector mutants have weak phenotypes, whereas polymutants lacking multiple T3Es show larger defects in bacterial growth or disease [12,19]. Such experiments demonstrate genetic buffering even when the relevant effectors act through different molecular targets.

AvrE1 and HopM1 illustrate the distinction between molecular mechanism and physiological convergence. AvrE1 acts through host phosphatase/ABA-associated processes, whereas HopM1 destabilizes MIN7 and perturbs trafficking; nevertheless, both are required for efficient formation of the aqueous apoplast that supports bacterial multiplication [57,58,59,67]. The evidence therefore supports functional convergence at the level of host water relations. It does not, by itself, establish direct AvrE1–HopM1 binding or a shared biochemical target.

### 4.2. Functional Specialization Enables Multilayer Host Manipulation

Specialization is supported by the diversity of validated DC3000 targets and subcellular sites. AvrPto/AvrPtoB act prominently at early immune perception and protein turnover; HopF2 targets MAPK signaling; HopM1 affects endomembrane trafficking; HopO1-1 alters plasmodesmatal communication; and HopI1, HopN1, and HopG1 perturb organelle-associated functions [14,15,16,17,18,54,55,56,57,58,59,60,61,62,63]. These activities represent experimentally separable routes into host biology rather than interchangeable versions of a single virulence function.

The functional consequence of this specialization is a distributed repertoire in which loss of one molecular route need not abolish infection if other essential host processes remain manipulated. Conversely, the same effector can have multiple consequences because host pathways are interconnected. Thus, “specialization” refers here to experimentally distinguishable mechanisms, not to an assumption that each effector has only one target or one biological effect.

### 4.3. Complementary and Cooperative Effects: Evidence and Limits of Interpretation

Cooperation should be reserved for cases in which a combinatorial experiment demonstrates that two or more effectors produce an outcome not explained by their individual activities. Shared effects on host physiology are insufficient on their own. In DC3000, the most informative evidence comes from reconstruction and pairwise-delivery systems in which effectors are introduced singly and in defined combinations [19,20].

AvrE1 and HopM1 are therefore described here as functionally convergent or complementary, not as a proven physically cooperative pair: both promote an aqueous apoplast through distinct mechanisms [57,58,59,67]. By contrast, the D36E effectorless background has enabled systematic single- and pairwise testing of the DC3000 repertoire, revealing combinations that suppress or modify phenotypes elicited by individual T3Es [20]. Such designs provide a stronger basis for claiming an effector–effector functional interaction because the relevant combination is tested directly.

### 4.4. Antagonistic Interactions and the Balance of Virulence Strategies

Antagonistic or metaeffector relationships are experimentally clearer when one effector modifies the host response elicited by another. A well-defined DC3000 example involves HopAD1 and AvrPtoB: in *N. benthamiana*, HopAD1 elicits immunity-associated cell death, whereas AvrPtoB suppresses this response in specified effector-delivery backgrounds [21]. This interaction should therefore be regarded as context-dependent, not as universally established across plant hosts or infection conditions. Pairwise repertoire screens likewise identified multiple DC3000 T3Es capable of suppressing cell-death phenotypes elicited by other effectors [20]; broader data support metaeffector action [38].

These findings also show why antagonism should not be inferred from evolutionary co-occurrence alone. The strongest claims require pairwise or combinatorial experiments in a defined bacterial background and host genotype. Where such evidence is absent, we use terms such as convergence, complementarity, or predicted linkage rather than direct antagonism.

### 4.5. Effector Networks as Emergent Properties of Pst DC3000 Virulence

Taken together, the DC3000 repertoire is best represented as a multilayer network with different evidentiary meanings. Molecular edges connect effectors to validated host targets; genetic edges denote compensation or epistasis measured by deletion/reconstruction; functional edges denote pairwise suppression or enhancement; and host-response or computational data can suggest additional associations that remain to be tested. This distinction prevents a network diagram from implying a level of molecular certainty that the underlying experiment does not provide. An evidence-defined network model explains why single deletions often have modest phenotypes, why polymutants can display severe defects, and why an effector may behave differently across hosts or delivery systems [12,19,20]. Table 3 summarizes representative DC3000 relationships together with the experimental basis and the boundary of interpretation.

### 4.6. Future Directions for Effector Network Analysis

The next step is to move from categorical interaction labels to quantitative epistasis. Future experiments should measure bacterial fitness, effector abundance/delivery, host genotype, cell type, infection stage, and environmental context within the same design. Combinatorial perturbation of defined effector sets, followed by preregistered statistical interaction tests, would distinguish additive effects from genuine synergy or antagonism. Single-cell and spatial readouts can then localize those interactions to host cellular states, while computational models can be evaluated against held-out genetic combinations rather than judged solely by network plausibility. These requirements are especially important as broader effectorome–NLRome models are developed [37].

## 5. Emerging Technologies Driving Systems-Level Understanding of *Pst* DC3000 Type III Effectors

Technological advances are most informative when their output is matched to the biological question being asked. Effectoromics tests many T3Es experimentally; multi-omics profiles infection-associated host states; single-cell and spatial methods resolve cellular heterogeneity; structural models generate hypotheses about folds and interfaces; and machine-learning classifiers prioritize candidate effectors or relationships. These methods therefore occupy different positions in the evidence chain and should not be grouped under a single label of “AI” or “systems biology”.

### 5.1. Effectoromics and High-Throughput Functional Screening

Effectoromics approaches have expanded the capacity to systematically investigate bacterial effectors by enabling large-scale analysis of effector–host relationships [68,69]. Compared with traditional studies centered on individual effectors, effectoromics allows comparative evaluation of diverse effectors and their effects on plant immunity, cellular processes, and infection phenotypes.

Transient expression systems, yeast-based functional assays, protein–protein interaction platforms, and large-scale genetic screening have been applied to identify host targets and define effector activities [69,70]. These studies have revealed that many *P. syringae* effectors converge on central host regulatory processes, including immune receptor signaling, protein kinase pathways, ubiquitination systems, and membrane trafficking machinery [70]. In parallel, effector polymutant analyses have provided critical evidence for understanding functional organization within the *Pst* DC3000 effector repertoire [12,19,20]. Systematic removal and subsequent reassembly of multiple effectors have demonstrated that virulence often depends on specific combinations of effectors rather than on individual components, revealing redundant effector groups and extensive functional interplay within the repertoire [12,19,20].

Effectoromics remains sensitive to experimental context. Heterologous overexpression can alter localization or dosage, and transient plant expression does not reproduce the timing and stoichiometry of native bacterial delivery. Comparisons using disarmed DC3000 backgrounds show that the surrounding effector repertoire can also change the phenotype of a delivered T3E [20]. Consequently, high-throughput hits should be retested with native or genetically defined T3SS delivery and infection-relevant expression levels.

### 5.2. Multi-Omics Approaches Reveal Host Responses to Effector Activity

Multi-omics technologies have provided a systems-level view of how *Pst* DC3000 infection and type III effector activity reshape host biology [71,72,73,74]. Transcriptomics, proteomics, metabolomics, and phosphoproteomics enable investigation of host responses across multiple molecular layers and facilitate connections between effector activities and broader cellular consequences.

Transcriptomic analyses have demonstrated extensive reprogramming of plant gene expression during *Pst* DC3000 infection, involving immune signaling, hormone regulation, metabolism, and stress-associated pathways [71]. Comparisons between wild-type bacteria and T3SS- or virulence-factor-deficient strains have further helped distinguish host transcriptional responses associated with bacterial virulence activities from basal immune responses [71]. Proteomic analyses provide complementary information by revealing changes in host protein abundance and secretion during *P. syringae* interactions [72], while phosphoproteomic approaches capture rapid alterations in signaling states that may not be apparent at the transcript level [73]. These approaches are particularly valuable because many T3Es alter protein activity, stability, localization, or post-translational modification without necessarily producing corresponding transcriptional changes. Metabolomic analyses further demonstrate extensive remodeling of host primary and secondary metabolism during *P. syringae* infection, linking pathogen activity to changes in nutrient allocation and physiological state [74].

Multi-omics data are powerful for identifying host states associated with infection, but changes in transcript abundance, phosphorylation, protein abundance, or metabolites do not establish a direct effector–target interaction. Mechanistic assignment requires orthogonal perturbation: effector deletion or complementation, target genetics, interaction/biochemical assays, localization, and phenotypic rescue where feasible. Omics is therefore best used to nominate pathways and quantify downstream consequences rather than to convert correlation into a molecular edge in an effector network.

### 5.3. Structural Biology and AI-Assisted Hypothesis Generation

Experimentally determined structures remain a direct source of mechanistic information. For example, structural studies on AvrPtoB–host kinase complexes have resolved effector–kinase recognition interfaces, while HopA1 structures provide a framework for testing functional hypotheses [75,76]. Structure prediction serves a different purpose: it can prioritize folds, domains, or candidate interfaces for proteins that lack experimentally determined structures, but the prediction itself is not evidence that a particular interaction occurs in planta.

AI-assisted tools in effector research should be separated by task. Sequence- and genome-context classifiers such as DeepT3 2.0, Effectidor, and the pan-genomic Effectidor II prioritize candidate T3Es [31,32,77]. Protein language-model approaches can add sequence representations to effector classification; recent cross-modal models such as CLEF illustrate the broader potential of combining language embeddings with biological features, although they were developed across bacterial secretion systems rather than specifically for *Pst* DC3000 [78]. AlphaFold-type models address protein structure, whereas complex prediction can prioritize candidate effector–host interfaces for experimental testing [26,30]. Network inference integrates these outputs with interaction and omics data but represents a further level of abstraction.

Each computational layer has characteristic failure modes. Training sets are incomplete and biased toward already characterized proteins; negative sets may contain undiscovered effectors; closely related sequences can inflate apparent cross-validation performance; and divergent pathovars may fall outside the training distribution. Structure and complex scores also do not report whether two proteins encounter one another at the correct place, time, abundance, or host genotype. Accordingly, false positives and uncertain predictions should be reported rather than hidden by a binary “AI-predicted” label.

### 5.4. Single-Cell and Spatial Technologies Reveal Cellular Heterogeneity During Infection

Plant tissues consist of diverse cell populations with distinct physiological states, and pathogen infection does not affect all host cells uniformly. Conventional bulk transcriptomic analyses provide averaged signals across tissues and therefore cannot fully resolve cell-type-specific immune responses.

Single-cell transcriptomic studies have now directly demonstrated substantial cellular heterogeneity in *Arabidopsis* leaves challenged with *Pseudomonas syringae*. Single-cell RNA sequencing revealed pathogen-responsive cell populations spanning immune and susceptible transcriptional states and showed that these states exhibit distinct temporal and spatial distributions during infection [28]. Independently, single-cell profiling of *Arabidopsis* leaves inoculated with *Pst* DC3000 identified characteristic responses in mesophyll, guard, epidermal, companion, and vascular cell populations, demonstrating extensive cell-type specialization and coordination during bacterial attack [79].

Spatial transcriptomics and advanced imaging technologies provide an important complementary dimension by preserving information regarding where pathogen-induced molecular changes occur within infected tissues. The integration of single-cell, single-nucleus, imaging, and spatial transcriptomic approaches is therefore expected to clarify how local tissue environments, intercellular communication, and proximity to pathogen colonization sites influence immune and susceptibility states [80]. Such approaches may eventually help determine whether individual effectors or effector combinations exert preferential effects in particular cellular contexts.

Although single-cell and spatial technologies remain technically challenging in plant–microbe interaction studies, their integration with effector biology is expected to provide a more refined understanding of how bacterial virulence is organized across cellular and spatial landscapes.

### 5.5. From Computational Prioritization to Experimentally Testable Predictions

A predictive framework should specify what is being predicted and how that prediction can fail. For DC3000, tractable endpoints include whether an open reading frame is a T3SS substrate, whether an effector localizes to a particular host compartment, whether a candidate host protein is required for an effector phenotype, whether two T3Es interact genetically, and whether an effector combination changes bacterial fitness. These endpoints can be evaluated experimentally and therefore provide stronger benchmarks than visually plausible network diagrams.

Genome-scale classifiers such as Effectidor/Effectidor II and DeepT3 2.0 can prioritize candidate effectors [31,32,77], while structure and protein-language-model methods can suggest functional families or candidate interfaces [30,33,78]. Host–pathogen network methods can then rank host proteins or pathways for testing [81,82]. The critical transition is from ranking to perturbation: a predicted target should be challenged genetically or biochemically, and a predicted effector–effector link should be tested in defined single and combinatorial delivery backgrounds.

**Figure 4:**
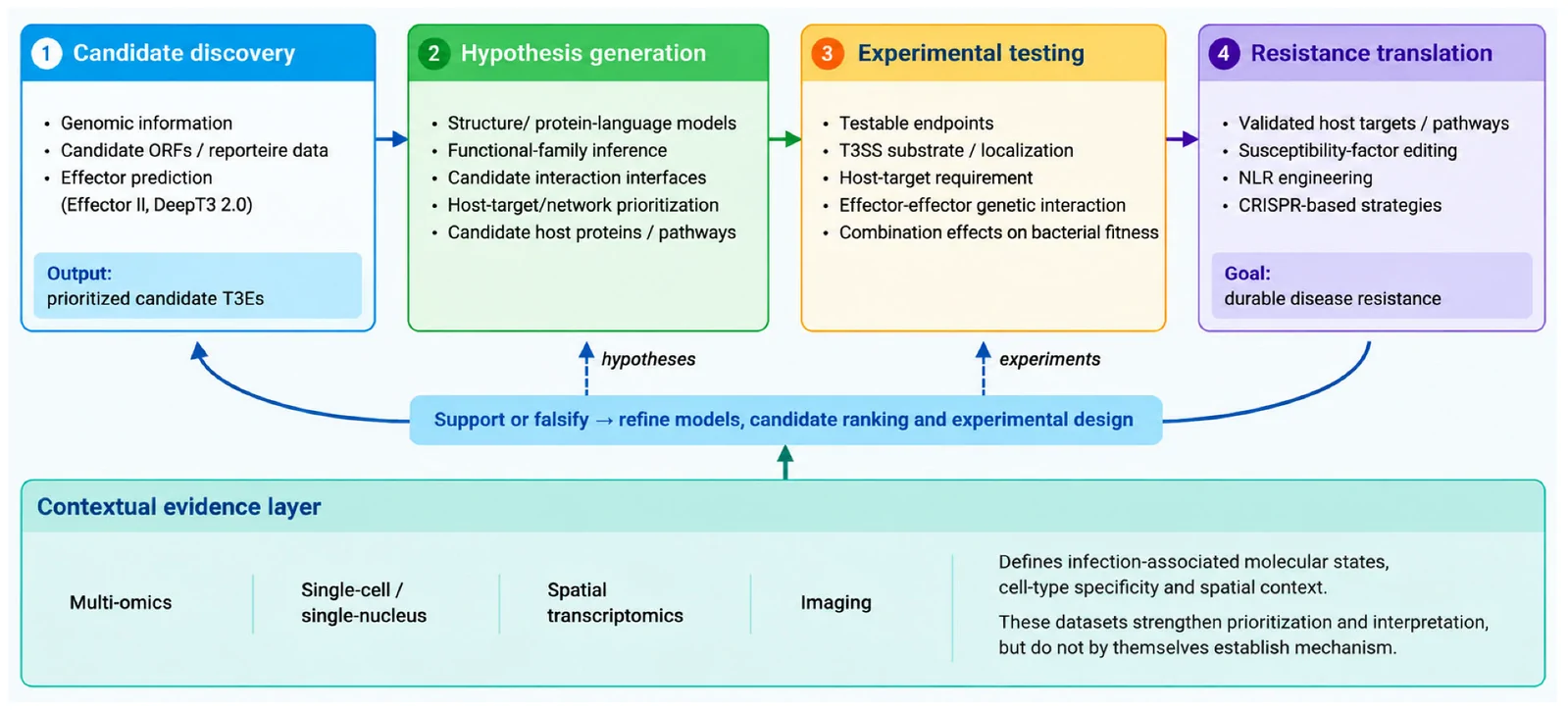
Integrated workflow for effector-guided discovery and translation of plant disease resistance. Candidate type III effectors (T3Es) are prioritized from genomic and repertoire data, followed by structure- and function-informed hypothesis generation, experimental validation of effector activities and host targets, and translation into resistance strategies. Multi-omics, single-cell/single-nucleus analyses, spatial transcriptomics, and imaging provide contextual evidence that supports candidate prioritization and mechanistic interpretation. Experimental outcomes iteratively refine models, candidate ranking, and subsequent experimental design.

The most useful integrated pipeline is therefore iterative: computational models nominate candidates; molecular and infection experiments falsify or support those candidates; and the resulting data are fed back into model development. This framework also makes uncertainty visible. It prevents AlphaFold or network inference from being presented as direct interaction evidence and provides a practical route for linking discovery to disease-resistance targets (Figure 4).

## 6. Future Perspectives: Toward Predictive Understanding of *Pst* DC3000 Effector-Mediated Virulence

Although substantial progress has been made in elucidating the molecular functions of *Pst* DC3000 type III effectors, many aspects of effector-mediated virulence remain unresolved. Current studies have revealed the activities of individual effectors and several functional relationships among them; however, how complex effector repertoires are coordinated during natural infection, how host cellular heterogeneity shapes effector activity, and how effector networks evolve under host immune selection remain poorly understood [45,83,84]. Recent comparative and evolutionary analyses further demonstrate that *P. syringae* effector repertoires are highly dynamic and are shaped by diversification, gain and loss, and host immune selection [35]. Future research will therefore require integration of molecular approaches with quantitative analyses and computational modeling to move *Pst* DC3000 effector biology beyond descriptive characterization toward predictive understanding of pathogen–host interactions.

### 6.1. Temporal and Spatial Regulation of Effector Deployment

A central challenge in understanding T3E-mediated pathogenicity is defining the temporal and spatial dynamics of effector activity during infection. Although many *Pst* DC3000 effectors have been characterized under controlled laboratory conditions, bacterial infection represents a dynamic process influenced by bacterial population changes, host immune status, nutrient availability, and environmental fluctuations. Expression of the *Pst* DC3000 type III secretion system and many effector genes is controlled by the HrpL regulatory network and is strongly influenced by environmental and metabolic signals [45,83,84]. Carbon sources, bacterial population density, and host-derived sugars can alter expression of *hrp* genes and individual effectors, indicating that effector deployment is tightly coupled to the local infection environment [45,84]. Different effectors may therefore contribute preferentially at distinct stages or locations during infection. Some may function primarily during initial immune suppression, whereas others may subsequently contribute to bacterial proliferation by altering host metabolism, nutrient availability, or cellular physiology. However, the mechanisms controlling effector expression, secretion, localization, and activity across infection stages remain incompletely understood.

Future integration of live-cell imaging, spatial transcriptomics, single-cell analysis, and quantitative proteomics should enable more precise investigation of how effector combinations are deployed within host tissues. Spatial transcriptomic approaches have already demonstrated that localized plant immune responses can be resolved across infected or elicited tissues [85], while single-cell studies increasingly reveal marked cell-type-specific heterogeneity in plant responses to biotic stress [86]. Extending these approaches to effector-resolved infection models may reveal whether particular effector modules are deployed sequentially or simultaneously and how their activities are coordinated during infection progression.

### 6.2. Quantitative Analysis and Modeling of Effector Networks

The discovery of effector redundancy, functional interaction, and convergence on host regulatory hubs has demonstrated that bacterial virulence cannot be adequately explained by simple one-effector–one-target models [87,88]. Large-scale host–pathogen interaction networks have shown that evolutionarily distinct pathogen effectors frequently converge on highly connected host proteins, supporting the concept that virulence emerges from perturbation of interconnected host regulatory networks rather than isolated molecular interactions [87,88].

A major challenge is therefore to determine how individual effectors quantitatively contribute to bacterial fitness, how regulatory nodes are organized within virulence networks, and how environmental conditions influence effector interactions during infection. Comparative analyses of the *P. syringae* effectorome further indicate that functional redundancy, diversification, horizontal transfer, and host immune recognition collectively shape the organization and evolution of effector repertoires [35]. Recent evolutionary reconstruction of the HopZ effector family has provided direct evidence that effector sequence diversification, altered expression, pseudogenization, and changes in host recognition can emerge during host–pathogen coevolution [89].

Addressing these questions will require integration of genetic perturbation approaches, large-scale phenotyping, and computational modeling. Effector polymutant libraries combined with machine learning and network-based analyses may enable systematic prediction of functional relationships among effectors and identification of key virulence modules. Deep-learning frameworks are already capable of identifying type III-secreted effectors at genome scale [32]. A future challenge will be to extend such computational approaches from effector identification toward prediction of functional relationships, genetic interactions, and infection phenotypes. Such quantitative frameworks could provide deeper insight into why pathogens maintain complex effector repertoires and how distinct effector combinations collectively shape infection outcomes.

### 6.3. Integrating Structural Biology, Artificial Intelligence, and Functional Validation

The immediate value of AI-assisted analysis is prioritization. Candidate effectors can be ranked by secretion-related features; structures can be modeled to identify conserved folds; and putative host interfaces can be proposed for mutational testing [30,32,33,77,78]. These tasks should remain analytically distinct because each has different training data, uncertainty, and experimental validation requirements. For plant-pathogen applications, validation must also preserve biological context. Host genotype, effector dosage, subcellular localization, infection stage, and the surrounding effector repertoire can all alter phenotype. A predicted complex therefore becomes mechanistically persuasive only when physical or biochemical evidence is combined with host genetics and an infection phenotype, preferably under native or defined T3SS delivery.

An iterative design—prediction, targeted perturbation, infection testing, and model revision—offers a more defensible route to predictive effector biology than one-time computational annotation. Structure-guided engineering of NLR immune receptors already illustrates how detailed interface knowledge can be converted into altered recognition specificity [90,91]. The same principle can be extended to bacterial pathosystems provided that model-derived hypotheses are subjected to independent validation.

### 6.4. Effector-Informed Strategies for Durable Disease Resistance

Effector biology has translational value when it identifies pathogen functions or host dependencies that are both important for disease and sufficiently constrained to limit escape. Classical ETI-based breeding exploits NLR recognition of individual effectors, but single recognition specificities can be eroded by effector loss, sequence change, altered expression, or metaeffector suppression [35,37,89,92]. Network information can therefore help prioritize effectors and host nodes that are conserved, genetically important, or targeted by multiple virulence activities.

One route is NLR stacking or engineering against core effectors whose loss carries a fitness cost [37,90,91,92]. A second route is to identify susceptibility (S) genes or effector-accessible host interfaces that can be modified without unacceptable developmental penalties. Reviews of bacterial plant diseases emphasize that S genes contribute to pathogen establishment, immune suppression, physiology, or symptom development, and that loss-of-susceptibility alleles can provide useful resistance phenotypes [93]. DC3000 effector-target maps can guide S-gene discovery, but agronomic value must be tested in relevant crops under agricultural conditions rather than inferred from *Arabidopsis* alone. These approaches also encourage combinations of resistance mechanisms. NLR engineering can improve recognition, while preserving host growth and reproduction. This distinction is essential when moving from mechanistic studies in *Arabidopsis* to durable resistance under agricultural conditions.

### 6.5. CRISPR-Based Functional Validation and Resistance Engineering

CRISPR technology provides both an experimental test of effector-network models and a route toward resistance engineering. In *Arabidopsis* or crop systems, CRISPR/Cas knockouts, promoter edits, base editing, or other precision approaches can be used to test whether a candidate susceptibility gene is required for a specific effector phenotype or for virulence by the intact pathogen. Where complete gene loss causes pleiotropic defects, editing an effector-binding surface or cis-regulatory element may preserve endogenous host function while reducing pathogen exploitation. Recent reviews highlight genome editing as a practical framework for identifying and deploying disease-resistance alleles [93,94].

Multiplex editing is particularly relevant to an effector-network perspective because several T3Es may converge on connected host processes. Editing multiple validated susceptibility nodes, or combining edited S genes with engineered/stacked NLRs, could in principle enhance resistance robustness. At present, however, this remains a research program rather than a field-validated DC3000-derived resistance strategy. Edited lines need evaluation for growth–defense trade-offs, pathogen spectrum, durability, environmental stability, and crop performance before claims of sustainable disease management are justified [93,94].

### 6.6. From Descriptive Effector Biology to Predictive Pathogen–Host Interaction Models

The longer-term goal is a model that predicts how a defined bacterial effector genotype behaves in a defined host genotype and environment. Building such models requires compatible measurements of effector sequence, expression and delivery, host targets, cell-type-specific responses, environmental conditions, and quantitative infection phenotypes. Multi-omics can contribute state variables, but model training and evaluation must be anchored to perturbation experiments [95].

Mechanistic and machine-learning models can then be evaluated prospectively: can they predict the phenotype of an unseen effector combination, a host target mutation, or a resistant genotype? Such held-out predictions provide a stronger test of “predictive biology” than retrospective fit to known interactions. They also create a direct bridge to breeding and genome-editing decisions because model errors become experimentally measurable. A mature DC3000 framework would therefore connect molecular causality, quantitative repertoire genetics, host-cell heterogeneity, and resistance engineering. Its contribution to sustainable agriculture will depend on whether those predictions remain valid in crop hosts and whether the resulting resistance reduces disease without unacceptable agronomic costs.

## 7. Conclusions

Research on *Pst* DC3000 has established unusually detailed links between individual bacterial effectors and host immune receptors, kinases, trafficking regulators, organelles, and physiological processes. The same system has also shown why those mechanisms cannot be interpreted in isolation: deletion, reconstruction, and pairwise-delivery experiments reveal genetic buffering, specialization, and context-dependent suppression within the repertoire. A rigorous network view must therefore preserve the distinction between a validated molecular interaction, a genetic interaction, physiological convergence, and a computationally inferred association.

Effectoromics, multi-omics, single-cell and spatial profiling, structure prediction, and machine-learning methods now expand the scale at which these questions can be addressed. Their principal contribution is to generate and prioritize testable hypotheses; they do not remove the need for native-delivery experiments, target genetics, biochemistry, and quantitative infection assays. Maintaining this evidence hierarchy is particularly important when results from *Arabidopsis*, tomato, *Nicotiana*, or other *P. syringae* pathovars are compared.

The translational challenge is to convert this mechanistic knowledge into resistance that remains effective under pathogen evolution and agricultural conditions. Conserved effector functions, susceptibility genes, engineered or stacked NLRs, and CRISPR-based editing provide complementary routes, but each must be assessed for durability, host fitness, pathogen spectrum, and field relevance. Progress toward sustainable crop protection will therefore depend less on accumulating additional predicted network edges than on closing the loop between prediction, experimental falsification, resistance engineering, and agronomic validation.

## Figures and Tables

**Table 1:** Positioning of the present review relative to selected prior syntheses and network-focused studies.

Primary Scope	Major Contribution	Boundary Addressed in the Present Review	Reference
DC3000 as a model pathogen	Integrated bacterial virulence, host immunity and physiology	Adds evidence-graded repertoire interactions and current resistance applications	Xin & He, 2013 [9]
Disarmed DC3000 polymutants	Defined key processes recoverable with a reduced effector set	Links reconstruction genetics to specific target mechanisms without equating convergence with direct cooperation	Wei & Collmer, 2018 [34]
Environmental regulation of the *P. syringae* T3SS	Synthesized host/environmental signals controlling HrpRS–HrpL deployment	Connects deployment context to downstream effector-network interpretation	O’Malley & Anderson, 2021 [36]
*P. syringae* effectorome diversity and evolution	Species-level synthesis of repertoire variation and evolutionary dynamics	Maintains a strain-centered DC3000 evidence framework	Bundalovic-Torma et al., 2022 [35]
Metaeffector control of ETI load	Experimentally demonstrated repertoire-dependent ETI suppression	Uses metaeffector data as a defined interaction class rather than a universal model	Martel et al., 2022 [38]
Effector biology in the AI era	Surveyed AI-enabled discovery and structural/function prediction	Separates discovery, structure, complex prediction and network inference, with validation requirements	Jones & Raffaele, 2025 [33]
Effectorome–NLRome network model	Interconnected modules, ETI load and resistance concepts	Provides DC3000-specific molecular examples and translational routes through S genes and genome editing	Xue et al., 2026 [37]

**Table 2:** Representative *Pst* DC3000 effectors, experimental systems, validated host targets or processes, and biological interpretations.

Effector	Strain/Host System	Validated Target or Process	Evidence Class	Biological Interpretation/Primary Refs
AvrPto	*Pst* DC3000; *Arabidopsis*/tomato	FLS2 and EFR kinase domains	Interaction, genetics, receptor signaling	Suppresses early PTI; recognized by Pto/Prf in resistant tomato [14,52]
AvrPtoB	*Pst* DC3000; *Arabidopsis*/*Nicotiana*	CERK1 and immune-protein turnover; E3 ligase activity	Biochemistry, interaction, infection genetics	Links receptor suppression to ubiquitin-dependent proteostasis [53,54]
HopF2	*Pst* DC3000; *Arabidopsis*	MKK5/MAPK signaling	ADP-ribosylation, signaling assays	Inhibits MAPK activation and downstream PTI [18]
HopAO1	*Pst* DC3000; *Arabidopsis*	EFR phosphorylation state	Protein tyrosine phosphatase assays	Attenuates EFR-dependent signaling [55]
HopM1	*Pst* DC3000; *Arabidopsis*	MIN7; endomembrane trafficking	Target degradation, genetics, localization	Perturbs defense trafficking and contributes to aqueous-apoplast formation [16,59,67]
AvrE1	*Pst* DC3000; *Arabidopsis*	TOPPs/ABA–SnRK2-associated water regulation	Biochemistry, genetics, physiology	Promotes stomatal closure and water-soaking [57,58]
HopO1-1	*Pst* DC3000; *Arabidopsis*	PDLP7 and potentially PDLP5 at plasmodesmata	Localization, interaction, stability assays	Increases intercellular movement and weakens immune-associated plasmodesmatal closure [60]
HopI1	*Pst* DC3000; *Arabidopsis*	Chloroplast Hsp70-associated processes	Localization, J-domain genetics, physiology	Remodels thylakoids and suppresses chloroplast-associated defense [17]
HopN1	*Pst* DC3000; *Arabidopsis*	PsbQ/photosystem II	Protease-target evidence, photosynthetic assays	Reduces PSII activity, ROS output and callose deposition [61]
HopG1	*Pst* DC3000; *Arabidopsis*	Mitochondrial physiology; actin-associated responses	Localization, respiration/ROS, cell biology	Connects mitochondrial perturbation with broader cellular reorganization [62,63]
HopAD1	*Pst* DC3000; *N. benthamiana* in disarmed backgrounds	MKK2-dependent immune/cell-death context	Pairwise delivery and genetic suppression	HopAD1-associated cell death is suppressed by AvrPtoB, providing a defined antagonistic interaction [21]

**Table 3:** Experimentally supported relationship classes within the *Pst* DC3000 effector repertoire.

Relationship Class	DC3000 Example	Experimental System	What the Evidence Supports	Primary Refs
Functional convergence/partially overlapping contributions	AvrE1 + HopM1	Deletion/reconstruction and *Arabidopsis* water-soaking phenotypes	Distinct molecular mechanisms make partially overlapping contributions to aqueous-apoplast formation; no direct physical interaction is implied	[57,58,59,67]
Genetic buffering	Polymutant/reduced-effector sets	Sequential deletions and reconstitution of disarmed DC3000	Virulence is distributed across partially overlapping modules; single-gene phenotypes can underestimate repertoire function	[12,19,34]
Antagonism/metaeffector action	HopAD1 ↔ AvrPtoB	Defined delivery in *N. benthamiana* using specified effector-delivery backgrounds	AvrPtoB suppresses HopAD1-associated cell death in this defined context; generality across hosts/conditions remains unestablished	[21]
Repertoire-level interplay	29 active T3Es in D36E background	Single and pairwise T3E delivery	Multiple T3Es suppress or modify phenotypes of other effectors; host/delivery context is critical	[20]
Specialization/complementarity	AvrPto/B, HopF2, HopM1, HopO1-1, HopI1/N1/G1	Target-specific biochemical, genetic and localization studies	Different effectors act through distinct host processes; pairwise synergy requires separate testing	[14,15,16,17,18,54,55,56,57,58,59,60,61,62,63]
ETI-load modulation (broader *P. syringae* evidence)	DC3000 repertoire in comparative ETI screens	*Arabidopsis*-based repertoire comparison	Effector background can suppress ETI elicited by other T3Es; supports metaeffector models beyond a single pair	[38]

## Data Availability

No new data were created or analyzed in this review article. All information presented herein was derived from previously published studies, which are appropriately cited throughout the manuscript. Figure 1, Figure 2, Figure 3 and Figure 4 are redrawn specifically for this revision using Python (version 3.12.3)/Matplotlib (version 3.9.2) from the authors’ synthesis of the cited literature; no panels or graphical elements were copied or adapted from published figures. No AI-assisted image-generation or image-editing tools were used in the preparation of Figure 1, Figure 2, Figure 3 and Figure 4. The revised diagrams explicitly distinguish experimentally supported relationships from predictive or conceptual links.

## References

[1] Jones J.D.G., Dangl J.L. (2006). The plant immune system. Nature.

[2] Dodds P.N., Rathjen J.P. (2010). Plant immunity: Towards an integrated view of plant–pathogen interactions. Nat. Rev. Genet..

[3] Snoeck S., Johanndrees O., Nürnberger T., Zipfel C. (2025). Plant pattern recognition receptors: From evolutionary insight to engineering. Nat. Rev. Genet..

[4] Yu X.-Q., Niu H.-Q., Liu C., Wang H.-L., Yin W., Xia X. (2024). PTI–ETI synergistic signal mechanisms in plant immunity. Plant Biotechnol. J..

[5] Jones J.D.G., Staskawicz B.J., Dangl J.L. (2024). The plant immune system: From discovery to deployment. Cell.

[6] Yu G., Macho A.P. (2026). Sabotage behind enemy lines: Bacterial hijacking of plant cellular functions. Curr. Opin. Plant Biol..

[7] Roussin-Léveillée C., Mackey D., Ekanayake G., Gohmann R., Moffett P. (2024). Extracellular niche establishment by plant pathogens. Nat. Rev. Microbiol..

[8] Figueroa M., Coaker G., Kanyuka K. (2024). Focus on the effectors at the interface of plant–microbe interactions. Mol. Plant Microbe Interact..

[9] Xin X.-F., He S.Y. (2013). *Pseudomonas syringae* pv. *tomato* DC3000: A model pathogen for probing disease susceptibility and hormone signaling in plants. Annu. Rev. Phytopathol..

[10] Buell C.R., Joardar V., Lindeberg M., Selengut J., Paulsen I.T., Gwinn M.L., Dodson R.J., Deboy R.T., Durkin A.S., Kolonay J.F. (2003). The complete genome sequence of the *Arabidopsis* and tomato pathogen *Pseudomonas syringae* pv. *tomato* DC3000. Proc. Natl. Acad. Sci. USA.

[11] Schechter L.M., Vencato M., Jordan K.L., Schneider S.E., Schneider D.J., Collmer A. (2006). Multiple approaches to a complete inventory of *Pseudomonas syringae* pv. *tomato* DC3000 type III secretion system effector proteins. Mol. Plant Microbe Interact..

[12] Kvitko B.H., Park D.H., Velásquez A.C., Wei C.-F., Russell A.B., Martin G.B., Schneider D.J., Collmer A. (2009). Deletions in the repertoire of *Pseudomonas syringae* pv. *tomato* DC3000 type III secretion effector genes reveal functional overlap among effectors. PLoS Pathog..

[13] Guo M., Tian F., Wamboldt Y., Alfano J.R. (2009). The majority of the type III effector inventory of *Pseudomonas syringae* pv. *tomato* DC3000 can suppress plant immunity. Mol. Plant Microbe Interact..

[14] Xiang T., Zong N., Zou Y., Wu Y., Zhang J., Xing W., Li Y., Tang X., Zhu L., Chai J. (2008). *Pseudomonas syringae* effector AvrPto blocks innate immunity by targeting receptor kinases. Curr. Biol..

[15] Göhre V., Spallek T., Häweker H., Mersmann S., Mentzel T., Boller T., de Torres M., Mansfield J.W., Robatzek S. (2008). Plant pattern-recognition receptor FLS2 is directed for degradation by the bacterial ubiquitin ligase AvrPtoB. Curr. Biol..

[16] Nomura K., Debroy S., Lee Y.H., Pumplin N., Jones J., He S.Y. (2006). A bacterial virulence protein suppresses host innate immunity to cause plant disease. Science.

[17] Jelenska J., Yao N., Vinatzer B.A., Wright C.M., Brodsky J.L., Greenberg J.T. (2007). A J domain virulence effector of *Pseudomonas syringae* remodels host chloroplasts and suppresses defenses. Curr. Biol..

[18] Wang Y., Li J., Hou S., Wang X., Li Y., Ren D., Chen S., Tang X., Zhou J.-M. (2010). A *Pseudomonas syringae* ADP-ribosyltransferase inhibits *Arabidopsis* mitogen-activated protein kinase kinases. Plant Cell.

[19] Cunnac S., Chakravarthy S., Kvitko B.H., Russell A.B., Martin G.B., Collmer A. (2011). Genetic disassembly and combinatorial reassembly identify a minimal functional repertoire of type III effectors in *Pseudomonas syringae*. Proc. Natl. Acad. Sci. USA.

[20] Wei H.-L., Zhang W., Collmer A. (2018). Modular study of the type III effector repertoire in *Pseudomonas syringae* pv. *tomato* DC3000 reveals a matrix of effector interplay in pathogenesis. Cell Rep..

[21] Wei H.-L., Chakravarthy S., Mathieu J., Helmann T.C., Stodghill P., Swingle B., Martin G.B., Collmer A. (2015). *Pseudomonas syringae* pv. *tomato* DC3000 type III secretion effector polymutants reveal an interplay between HopAD1 and AvrPtoB. Cell Host Microbe.

[22] Nobori T., Velásquez A.C., Wu J., Kvitko B.H., Kremer J.M., Wang Y., He S.Y., Tsuda K. (2018). Transcriptome landscape of a bacterial pathogen under plant immunity. Proc. Natl. Acad. Sci. USA.

[23] Nobori T., Wang Y., Wu J., Stolze S.C., Tsuda Y., Finkemeier I., Nakagami H., Tsuda K. (2020). Multidimensional gene regulatory landscape of a bacterial pathogen in plants. Nat. Plants.

[24] Kang J., Sokolowska E.M., Zillmer H., Karasov T., Walther D., Skirycz A. (2026). Discovery of regulatory complexes in *Arabidopsis* via protein–metabolite mapping during Pseudomonas infection. Plant Physiol..

[25] Seong K., Krasileva K.V. (2023). Prediction of effector protein structures from fungal phytopathogens enables evolutionary analyses. Nat. Microbiol..

[26] Homma F., Huang J., van der Hoorn R.A.L. (2023). AlphaFold-Multimer predicts cross-kingdom interactions at the plant–pathogen interface. Nat. Commun..

[27] Yu D.S., Outram M.A., Smith A., McCombe C.L., Khambalkar P.B., Rima S.A., Sun X., Ma L., Ericsson D.J., Jones D.A. (2024). The structural repertoire of *Fusarium oxysporum* f. sp. *lycopersici* effectors revealed by experimental and computational studies. eLife.

[28] Zhu J., Lolle S., Tang A., Guel B., Kvitko B., Cole B., Coaker G. (2023). Single-cell profiling of *Arabidopsis* leaves to *Pseudomonas syringae* infection. Cell Rep..

[29] Nobori T., Monell A., Lee T.A., Sakata Y., Shirahama S., Zhou J., Nery J.R., Mine A., Ecker J.R. (2025). A rare PRIMER cell state in plant immunity. Nature.

[30] Jumper J., Evans R., Pritzel A., Green T., Figurnov M., Ronneberger O., Tunyasuvunakool K., Bates R., Žídek A., Potapenko A. (2021). Highly accurate protein structure prediction with AlphaFold. Nature.

[31] Wagner N., Avram O., Gold-Binshtok D., Zerah B., Teper D., Pupko T. (2022). Effectidor: An automated machine-learning-based web server for the prediction of type-III secretion system effectors. Bioinformatics.

[32] Jing R., Wen T., Liao C., Xue L., Liu F., Yu L., Luo J. (2021). DeepT3 2.0: Improving type III secreted effector predictions by an integrative deep learning framework. NAR Genom. Bioinform..

[33] Jones D.A.B., Raffaele S. (2025). Phytopathogen effector biology in the burgeoning AI era. Annu. Rev. Phytopathol..

[34] Wei H.-L., Collmer A. (2018). Defining essential processes in plant pathogenesis with *Pseudomonas syringae* pv. *tomato* DC3000 disarmed polymutants and a subset of key type III effectors. Mol. Plant Pathol..

[35] Bundalovic-Torma C., Lonjon F., Desveaux D., Guttman D.S. (2022). Diversity, evolution, and function of *Pseudomonas syringae* effectoromes. Annu. Rev. Phytopathol..

[36] O’Malley M.R., Anderson J.C. (2021). Regulation of the *Pseudomonas syringae* type III secretion system by host environment signals. Microorganisms.

[37] Xue B., Guttman D.S., Ruan L. (2026). From gene-for-gene to NLRome-for-effectorome: Decoding the dynamic interplay between phytopathogenic effector networks and plant immune systems. FEMS Microbiol. Rev..

[38] Martel A., Laflamme B., Breit-McNally C., Wang P., Lonjon F., Desveaux D., Guttman D.S. (2022). Metaeffector interactions modulate the type III effector-triggered immunity load of *Pseudomonas syringae*. PLoS Pathog..

[39] Kvitko B.H., Collmer A. (2023). Discovery of the Hrp type III secretion system in phytopathogenic bacteria: How investigation of hypersensitive cell death in plants led to a novel protein injector system and a world of inter-organismal molecular interactions within plant cells. Phytopathology.

[40] Alfano J.R., Charkowski A.O., Deng W.-L., Badel J.L., Petnicki-Ocwieja T., van Dijk K., Collmer A. (2000). The *Pseudomonas syringae* Hrp pathogenicity island has a tripartite mosaic structure composed of a cluster of type III secretion genes bounded by exchangeable effector and conserved effector loci that contribute to parasitic fitness and pathogenicity in plants. Proc. Natl. Acad. Sci. USA.

[41] Wei W., Plovanich-Jones A., Deng W.-L., Jin Q.-L., Collmer A., Huang H.-C., He S.Y. (2000). The gene coding for the Hrp pilus structural protein is required for type III secretion of Hrp and Avr proteins in *Pseudomonas syringae* pv. *Tomato*. Proc. Natl. Acad. Sci. USA.

[42] Jin Q., He S.Y. (2001). Role of the Hrp pilus in type III protein secretion in *Pseudomonas syringae*. Science.

[43] Lan L., Deng X., Zhou J., Tang X. (2006). Genome-wide gene expression analysis of *Pseudomonas syringae* pv. *tomato* DC3000 reveals overlapping and distinct pathways regulated by hrpL and hrpRS. Mol. Plant Microbe Interact..

[44] Haapalainen M., van Gestel K., Pirhonen M., Taira S. (2009). Soluble plant cell signals induce the expression of the type III secretion system of *Pseudomonas syringae* and upregulate the production of pilus protein HrpA. Mol. Plant Microbe Interact..

[45] Stauber J.L., Loginicheva E., Schechter L.M. (2012). Carbon source and cell density-dependent regulation of type III secretion system gene expression in *Pseudomonas syringae* pathovar *tomato* DC3000. Res. Microbiol..

[46] Yan Q., Rogan C.J., Pang Y.-Y., Davis E.W., Anderson J.C. (2020). Ancient co-option of an amino acid ABC transporter locus in *Pseudomonas syringae* for host signal-dependent virulence gene regulation. PLoS Pathog..

[47] Lindeberg M., Cunnac S., Collmer A. (2012). *Pseudomonas syringae* type III effector repertoires: Last words in endless arguments. Trends Microbiol..

[48] Dillon M.M., Almeida R.N.D., Laflamme B., Martel A., Weir B.S., Desveaux D., Guttman D.S. (2019). Molecular evolution of *Pseudomonas syringae* type III secreted effector proteins. Front. Plant Sci..

[49] Rohmer L., Guttman D.S., Dangl J.L. (2004). Diverse evolutionary mechanisms shape the type III effector virulence factor repertoire in the plant pathogen *Pseudomonas syringae*. Genetics.

[50] Baltrus D.A., Nishimura M.T., Romanchuk A., Chang J.H., Mukhtar M.S., Cherkis K., Roach J., Grant S.R., Jones C.D., Dangl J.L. (2011). Dynamic evolution of pathogenicity revealed by sequencing and comparative genomics of 19 *Pseudomonas syringae* isolates. PLoS Pathog..

[51] Munkvold K.R., Russell A.B., Kvitko B.H., Collmer A. (2009). *Pseudomonas syringae* pv. *tomato* DC3000 type III effector HopAA1-1 functions redundantly with chlorosis-promoting factor PSPTO4723 to produce bacterial speck lesions in host tomato. Mol. Plant Microbe Interact..

[52] Xiang T., Zong N., Zhang J., Chen J., Chen M., Zhou J.-M. (2011). BAK1 is not a target of the *Pseudomonas syringae* effector AvrPto. Mol. Plant Microbe Interact..

[53] Janjusevic R., Abramovitch R.B., Martin G.B., Stebbins C.E. (2006). A bacterial inhibitor of host programmed cell death defenses is an E3 ubiquitin ligase. Science.

[54] Gimenez-Ibanez S., Hann D.R., Ntoukakis V., Petutschnig E., Lipka V., Rathjen J.P. (2009). AvrPtoB targets the LysM receptor kinase CERK1 to promote bacterial virulence on plants. Curr. Biol..

[55] Macho A.P., Schwessinger B., Ntoukakis V., Brutus A., Segonzac C., Roy S., Kadota Y., Oh M.-H., Sklenar J., Derbyshire P. (2014). A bacterial tyrosine phosphatase inhibits plant pattern recognition receptor activation. Science.

[56] Kim S.H., Kwon S.I., Saha D., Anyanwu N.C., Gassmann W. (2009). Resistance to the *Pseudomonas syringae* effector HopA1 is governed by the TIR-NBS-LRR protein RPS6 and is enhanced by mutations in SRFR1. Plant Physiol..

[57] Roussin-Léveillée C., Lajeunesse G., St-Amand M., Pillay Veerapen V., Silva-Martins G., Nomura K., Brassard S., Bolaji A., He S.Y., Moffett P. (2022). Evolutionarily conserved bacterial effectors hijack abscisic acid signaling to induce an aqueous environment in the apoplast. Cell Host Microbe.

[58] Hu Y., Ding Y., Cai B., Qin X., Wu J., Yuan M., Wan S., Zhao Y., Xin X.-F. (2022). Bacterial effectors manipulate plant abscisic acid signaling for creation of an aqueous apoplast. Cell Host Microbe.

[59] Nomura K., Mecey C., Lee Y.-N., Imboden L.A., Chang J.H., He S.Y. (2011). Effector-triggered immunity blocks pathogen degradation of an immunity-associated vesicle traffic regulator in *Arabidopsis*. Proc. Natl. Acad. Sci. USA.

[60] Aung K., Kim P., Li Z., Joe A., Kvitko B., Alfano J.R., He S.Y. (2020). Pathogenic bacteria target plant plasmodesmata to colonize and invade surrounding tissues. Plant Cell.

[61] Rodríguez-Herva J.J., González-Melendi P., Cuartas-Lanza R., Antúnez-Lamas M., Río-Alvarez I., Li Z., López-Torrejón G., Díaz I., del Pozo J.C., Chakravarthy S. (2012). A bacterial cysteine protease effector protein interferes with photosynthesis to suppress plant innate immune responses. Cell. Microbiol..

[62] Block A., Guo M., Li G., Elowsky C., Clemente T.E., Alfano J.R. (2010). The *Pseudomonas syringae* type III effector HopG1 targets mitochondria, alters plant development and suppresses plant innate immunity. Cell. Microbiol..

[63] Shimono M., Lu Y.-J., Porter K., Kvitko B.H., Henty-Ridilla J., Creason A., He S.Y., Chang J.H., Staiger C.J., Day B. (2016). The *Pseudomonas syringae* type III effector HopG1 induces actin remodeling to promote symptom development and susceptibility during infection. Plant Physiol..

[64] Zheng X.-Y., Weaver Spivey N., Zeng W., Liu P.-P., Fu Z.Q., Klessig D.F., He S.Y., Dong X. (2012). Coronatine promotes *Pseudomonas syringae* virulence in plants by activating a signaling cascade that inhibits salicylic acid accumulation. Cell Host Microbe.

[65] Jiang S., Yao J., Ma K.-W., Zhou H., Song J., He S.Y., Ma W. (2013). Bacterial effector activates jasmonate signaling by directly targeting JAZ transcriptional repressors. PLoS Pathog..

[66] Gimenez-Ibanez S., Boter M., Fernández-Barbero G., Chini A., Rathjen J.P., Solano R. (2014). The bacterial effector HopX1 targets JAZ transcriptional repressors to activate jasmonate signaling and promote infection in *Arabidopsis*. PLoS Biol..

[67] Xin X.-F., Nomura K., Aung K., Velásquez A.C., Yao J., Boutrot F., Chang J.H., Zipfel C., He S.Y. (2016). Bacteria establish an aqueous living space in plants crucial for virulence. Nature.

[68] Cunnac S., Lindeberg M., Collmer A. (2009). *Pseudomonas syringae* type III secretion system effectors: Repertoires in search of functions. Curr. Opin. Microbiol..

[69] Munkvold K.R., Martin M.E., Bronstein P.A., Collmer A. (2008). A survey of the *Pseudomonas syringae* pv. *tomato* DC3000 type III secretion system effector repertoire reveals several effectors that are deleterious when expressed in *Saccharomyces cerevisiae*. Mol. Plant Microbe Interact..

[70] Block A., Alfano J.R. (2011). Plant targets for *Pseudomonas syringae* type III effectors: Virulence targets or guarded decoys?. Curr. Opin. Microbiol..

[71] Thilmony R., Underwood W., He S.Y. (2006). Genome-wide transcriptional analysis of the *Arabidopsis thaliana* interaction with the plant pathogen *Pseudomonas syringae* pv. *tomato* DC3000 and the human pathogen *Escherichia coli* O157:H7. Plant J..

[72] Kaffarnik F.A.R., Jones A.M.E., Rathjen J.P., Peck S.C. (2009). Effector proteins of the bacterial pathogen *Pseudomonas syringae* alter the extracellular proteome of the host plant, *Arabidopsis thaliana*. Mol. Cell. Proteom..

[73] Jones A.M.E., Bennett M.H., Mansfield J.W., Grant M. (2006). Analysis of the defence phosphoproteome of *Arabidopsis thaliana* using differential mass tagging. Proteomics.

[74] Ward J.L., Forcat S., Beckmann M., Bennett M., Miller S.J., Baker J.M., Hawkins N.D., Vermeer C.P., Lu C., Lin W. (2010). The metabolic transition during disease following infection of *Arabidopsis thaliana* by *Pseudomonas syringae* pv. *tomato*. Plant J..

[75] Cheng W., Munkvold K.R., Gao H., Mathieu J., Schwizer S., Wang S., Yan Y.-B., Wang J., Martin G.B., Chai J. (2011). Structural analysis of *Pseudomonas syringae* AvrPtoB bound to host BAK1 reveals two similar kinase-interacting domains in a type III effector. Cell Host Microbe.

[76] Park Y., Shin I., Rhee S. (2015). Crystal structure of the effector protein HopA1 from *Pseudomonas syringae*. J. Struct. Biol..

[77] Wagner N., Baumer E., Lyubman I., Shimony Y., Bracha N., Martins L., Potnis N., Chang J.H., Teper D., Koebnik R. (2025). Effectidor II: A pan-genomic AI-based algorithm for the prediction of type III secretion system effectors. Bioinformatics.

[78] Peng Y., Wu J., Sun Y., Zhang Y., Wang Q., Shao S. (2025). Contrastive-learning of language embedding and biological features for cross modality encoding and effector prediction. Nat. Commun..

[79] Delannoy E., Batardiere B., Pateyron S., Soubigou-Taconnat L., Chiquet J., Colcombet J., Lang J. (2023). Cell specialization and coordination in *Arabidopsis* leaves upon pathogenic attack revealed by scRNA-seq. Plant Commun..

[80] Zhu J., Moreno-Pérez A., Coaker G. (2023). Understanding plant pathogen interactions using spatial and single-cell technologies. Commun. Biol..

[81] Sahu S.S., Weirick T., Kaundal R. (2014). Predicting genome-scale *Arabidopsis*–*Pseudomonas syringae* interactome using domain and interolog-based approaches. BMC Bioinform..

[82] Li H., Zhou Y., Zhang Z. (2017). Network analysis reveals a common host–pathogen interaction pattern in *Arabidopsis* immune responses. Front. Plant Sci..

[83] Ferreira A.O., Myers C.R., Gordon J.S., Martin G.B., Vencato M., Collmer A., Wehling M.D., Alfano J.R., Moreno-Hagelsieb G., Lamboy W.F. (2006). Whole-genome expression profiling defines the HrpL regulon of *Pseudomonas syringae* pv. *tomato* DC3000, allows de novo reconstruction of the Hrp *cis* element, and identifies novel coregulated genes. Mol. Plant Microbe Interact..

[84] Turner S.E., Pang Y.-Y., O’Malley M.R., Weisberg A.J., Fraser V.N., Yan Q., Chang J.H., Anderson J.C. (2020). A DeoR-type transcription regulator is required for sugar-induced expression of type III secretion-encoding genes in *Pseudomonas syringae* pv. *tomato* DC3000. Mol. Plant Microbe Interact..

[85] Giolai M., Verweij W., Lister A., Heavens D., Macaulay I., Clark M.D. (2019). Spatially resolved transcriptomics reveals plant host responses to pathogens. Plant Methods.

[86] Tenorio Berrío R., Dubois M. (2024). Single-cell transcriptomics reveals heterogeneity in plant responses to the environment: A focus on biotic and abiotic interactions. J. Exp. Bot..

[87] Mukhtar M.S., Carvunis A.-R., Dreze M., Epple P., Steinbrenner J., Moore J., Tasan M., Galli M., Hao T., Nishimura M.T. (2011). Independently evolved virulence effectors converge onto hubs in a plant immune system network. Science.

[88] Weßling R., Epple P., Altmann S., He Y., Yang L., Henz S.R., McDonald N., Wiley K., Bader K.C., Gläßer C. (2014). Convergent targeting of a common host protein-network by pathogen effectors from three kingdoms of life. Cell Host Microbe.

[89] McTavish K.J., Chepsergon J., Voitenko O., Wang P.W., Desveaux D., Guttman D.S. (2026). Signatures of the coevolutionary arms race in response to effector-triggered immunity in the *Pseudomonas syringae* HopZ type III effector family. Cell Rep..

[90] Zdrzałek R., Xi Y., Langner T., Bentham A.R., Petit-Houdenot Y., De la Concepcion J.C., Harant A., Shimizu M., Were V., Talbot N.J. (2024). Bioengineering a plant NLR immune receptor with a robust binding interface toward a conserved fungal pathogen effector. Proc. Natl. Acad. Sci. USA.

[91] Zdrzałek R., Stone C., De la Concepcion J.C., Banfield M.J., Bentham A.R. (2023). Pathways to engineering plant intracellular NLR immune receptors. Curr. Opin. Plant Biol..

[92] Zhang M., Coaker G. (2017). Harnessing effector-triggered immunity for durable disease resistance. Phytopathology.

[93] Baruah S., Hutin M., Bogdanove A.J. (2025). Susceptibility genes in bacterial diseases of plants. Annu. Rev. Phytopathol..

[94] Park H.J., Kim M., Lee D., Kim H.J., Jung H.W. (2024). CRISPR-Cas9 and beyond: Identifying target genes for developing disease-resistant plants. Plant Biol..

[95] Balotf S., Wilson R., Hemmati R., Eshaghi M., Wilson C., Mur L.A.J. (2025). Plant–pathogen interactions: Making the case for multi-omics analysis of complex pathosystems. Stress Biol..

